# Auxiliary Segmentation Method of Osteosarcoma MRI Image Based on Transformer and U-Net

**DOI:** 10.1155/2022/9990092

**Published:** 2022-11-14

**Authors:** Feng Liu, Jun Zhu, Baolong Lv, Lei Yang, Wenyan Sun, Zhehao Dai, Fangfang Gou, Jia Wu

**Affiliations:** ^1^School of Information Engineering, Shandong Youth University of Political Science, Jinan, Shandong, China; ^2^New Technology Research and Development Center of Intelligent Information Controlling in Universities of Shandong, Jinan 250103, China; ^3^The First People's Hospital of Huaihua, Huaihua 418000, Hunan, China; ^4^Collaborative Innovation Center for Medical Artificial Intelligence and Big Data Decision Making Assistance, Hunan University of Medicine, Huaihua 418000, Hunan, China; ^5^School of Modern Service Management, Shandong Youth University of Political Science, Jinan, China; ^6^School of Computer Science and Technology, Shandong Janzhu University, Jinan, China; ^7^Department of Spine Surgery, The Second Xiangya Hospital, Central South University, Changsha 410011, China; ^8^School of Computer Science and Engineering, Central South University, Changsha 410083, China; ^9^Research Center for Artificial Intelligence, Monash University, Melbourne, Clayton, Victoria 3800, Australia

## Abstract

One of the most prevalent malignant bone tumors is osteosarcoma. The diagnosis and treatment cycle are long and the prognosis is poor. It takes a lot of time to manually identify osteosarcoma from osteosarcoma magnetic resonance imaging (MRI). Medical image processing technology has greatly alleviated the problems faced by medical diagnoses. However, MRI images of osteosarcoma are characterized by high noise and blurred edges. The complex features increase the difficulty of lesion area identification. Therefore, this study proposes an osteosarcoma MRI image segmentation method (OSTransnet) based on Transformer and U-net. This technique primarily addresses the issues of fuzzy tumor edge segmentation and overfitting brought on by data noise. First, we optimize the dataset by changing the precise spatial distribution of noise and the data-increment image rotation process. The tumor is then segmented based on the model of U-Net and Transformer with edge improvement. It compensates for the limitations of U-semantic Net by using channel-based transformers. Finally, we also add an edge enhancement module (BAB) and a combined loss function to improve the performance of edge segmentation. The method's accuracy and stability are demonstrated by the detection and training results based on more than 4,000 MRI images of osteosarcoma, which also demonstrate how well the method works as an adjunct to clinical diagnosis and treatment.

## 1. Introduction

Osteosarcoma is the most common primary malignant bone tumor, accounting for approximately 44% of primary malignant tumors in orthopedics [1]. In developing countries, limited by medical level, the death rate of osteosarcoma has far exceeded that of developed countries. The survival rate of patients with advanced osteosarcoma is less than 20% [2]. Early detection and timely development of reasonable treatment strategies can effectively improve the survival rate of patients [3]. The advantage of MRI is that it can detect aberrant signals in the early stages of a lesion. It can produce multidimensional images thanks to its multidirectional imaging. It can also display more information about the soft tissues and their links to the surrounding neurovascular [4]. It can also quantify the extent of the bone marrow cavity's involvement [5]. As a result, MRI is a critical technique for doctors to use when diagnosing and evaluating probable osteosarcoma.

In most developing countries, the treatment and prognosis of osteosarcoma have been troubling for those involved, and it is also a pain point for every osteosarcoma patient. Developing nations are unable to provide patients with osteosarcoma with a more individualized course of treatment due to their economic underdevelopment and lack of medical resources and equipment [6]. On the other hand, the lack of technical personnel and the backward medical technology make the early diagnosis of osteosarcoma a huge problem [7–10]. The larger problem is that even with adequate screening equipment and MRI images, inefficient manual recognition measures may lead to delays in diagnosis and treatment, thus worsening the condition of patients with osteosarcoma. Since 600–700 MRI images are generated per patient [11], there are often fewer than 20 valid osteosarcoma images. A large amount of data can only be diagnosed by doctors' manual identification [11, 12], which burdens doctors. Long-term high-intensity work can also fatigue doctors and reduce the speed and accuracy of discrimination [13]. Worst of all, the location, structure, shape, and density of different osteosarcomas are not identical [14]. It is difficult to distinguish the tumor location from normal tissues. Different osteosarcomas may also have image differences under the same imaging method [15–17]. It is extremely difficult to diagnose with the naked eye, which requires doctors to have rich diagnostic experience. Otherwise, it may lead to inaccurate diagnostic results and delays in patient treatment [18].

Medical image processing technology has steadily been employed in the direction of medical diagnostics as computer image technology has progressed [19]. Among the existing studies, there are many types of segmentation algorithms applied to medical images, such as thresholding [20, 21], region growing [22, 23], machine learning [24, 25], deep learning [26, 27], active contouring [28, 29], quantum-inspired compilation [30, 31], and computational intelligence [32, 33]. These algorithms are able to provide effective support for the clinical routine. Through algorithm processing, the system can more accurately segment the tumor area that the doctor is interested in [34]. It is helpful for precise localization and diagnosis and treatment, reducing the possibility of tumor recurrence, and thereby greatly improving the survival rate of patients [35]. For example, the literature [36] uses the convolutional neural network for the localization and segmentation of brain tumors, and the literature [37] realizes the classification of brain tumors and the grading of glial tumors. However, segmenting osteosarcoma MRI images remains a significant difficulty. The amount of noise in MRI pictures varies. Furthermore, the segmentation model is prone to noise [38] and overfitting, resulting in worse segmentation accuracy. Meanwhile, osteosarcoma has a wide range of local tissue development and shape [39, 40]. These properties cause indistinct tumor boundaries and complex form structures, making it difficult to maintain edge features [41–43]. As a result, it is worth looking into how to segment osteosarcoma effectively and properly.

We present a segmentation approach for osteosarcoma MRI images using edge enhancement features (OSTransnet). To begin, we optimize the dataset by altering the spatial distribution of natural noise. The overfitting problem of deep learning models caused by MRI image noise is solved using this method. Then, for osteosarcoma image segmentation (UCTransnet), we employed Transformer and U-net network models. The channel CTrans module was introduced by UCTransnet. The jump connection element of U-Net is replaced by this module. This method compensates for U-Net segmentation's semantic shortcomings and accomplishes global multiscale segmentation of tumor patches of various sizes. This approach also increases the accuracy of osteosarcoma segmentation by resolving complicated and changeable lesion areas in MRI images of osteosarcoma. Finally, we employ a combined loss function and an edge augmentation module. They collaborate to improve the segmentation results and effectively handle the problem of tumor edge blurring. This method increases diagnostic efficiency while reducing diagnostic workload and time without compromising diagnostic accuracy.

The contributions to this paper are listed as follows:

1. A new data alignment method is introduced in this paper to optimize the dataset. The new data alignment is achieved by altering the spatial distribution of real noise to generate more training samples that include both actual content and noise. The strategy effectively mitigates the effect of noise on model segmentation while broadening the data.
2. The segmentation model utilized in this paper is UCTransnset, which is built on Transformer and U-Net. Instead of using the skip-connected section of the U-Net, this network structure uses the channelized Transformer module (CTrans). It realizes the localization and identification of tumors of different scales.
3. The edge enhancement module (BAB) with a combined loss function is introduced in this study. This module can increase tumor border segmentation accuracy and effectively tackle the problem of tumor edge blurring.
4. The experimental results show that our proposed method of osteosarcoma segmentation has higher precision than previous methods and has advantages in various evaluation indexes. The results can be used by physicians to assist in the diagnosis and treatment of osteosarcoma. This study has important implications for the ancillary diagnosis, treatment, and prognosis of osteosarcoma.

## 2. Related Work

With the development of computer technology, there have been many artificial intelligence decision-making systems and image processing methods used in these systems to assist in disease diagnosis. In the diagnosis of osteosarcoma, we use computer technology to analyze and process images to help doctors quickly find the tumor location and improve the speed and accuracy of diagnosis. This has become a research hotspot today, and some mainstream algorithms in this field are introduced below:

To discriminate between live tumors, necrotic tumors, and nontumors, Ahmed et al. [44] proposed a compact CNN architecture to classify osteosarcoma images. The method combines a regularized model with the CNN architecture to reduce overfitting, which achieves good results on balanced datasets. Fu et al. [45] designed a DS-Net algorithm combining a depth model with a Siamese network to address the phenomenon of overfitting of small datasets in osteosarcoma classification. Anisuzzaman et al. [46] used a CNN network for pretraining. In this way, an automatic classifier of osteosarcoma tissue images is realized, thereby better predicting the patient's condition.

Additionally, a lot of research has suggested osteosarcoma segmentation algorithms that predict and separate the tumor region of osteosarcoma. Nasir and Obaid [47] proposed an algorithm-KCG that combines multiple image processing techniques, which involves iterative morphological operations and object counting, and achieves high accuracy on existing datasets. The MSFCN method was proposed by Huang et al. [48]. The idea is to add a supervised output layer to ensure that both local and global image features can be captured. The MSRN proposed by Zhang et al. [49] can provide automatic and accurate segmentation for the osteosarcoma region of the image. By adding three additional supervised side output modules, the extraction of image shape and semantic features is realized respectively. Shuai et al. [50] designed a W-net++ model by considering two cascading U-Net networks in an integrated manner. It is mainly implemented by applying multiscale inputs to the network and introducing deep adaptive supervision. Ho et al. [51] described a deeply interactive learning (DIAL) approach to training a CNN as a labeling method for predictive assessment of prognostic factors for survival in osteosarcoma. This method can effectively predict the necrosis rate within the variation rate range.

In addition to its use for osteosarcoma segmentation, there are many studies on the application of computer technology in the treatment of osteosarcoma. Kim et al. [52] compared the performance of different methods in predicting response to neoadjuvant chemotherapy in osteosarcoma patients, which can help clinicians, decide whether to proceed with further treatment of this patient. Dufau et al. [53] developed a support vector machine-based predictive model to predict the treatment effect of neoadjuvant chemotherapy, which predicted the chemotherapy response of patients before starting treatment. Hu et al. [46, 54] established an MRI image recognition model based on the proposed CSDCNN algorithm. This method obtained better indicators than SegNet, LeNet, and other algorithms. The F-HHO-based GAN proposed by Badshah et al. [47, 54] can be used for early osteosarcoma detection work. The method classifies tumors by GAN and uses GAN to detect and segment the extracted image features.

With the development of deep learning-based networks, many researchers embed the latest algorithms of the team into the system for implementation. Arunachalam et al. [55] created a deep learning architecture that implements a fully automated tumor classification system. It establishes the groundwork for automating the deep learning algorithms' extraction of tumor prediction maps from raw images. Bansal et al. [56] implemented an automatic detection system based on the F-FSM-C classification model. The model can classify the original image into three types: surviving tumor, nonsurviving tumor, and nontumor, reducing the number of network features. In view of the characteristic of high noise in osteosarcoma MRI images, Wu et al. [57] proposed a segmentation system based on deep convolutional neural networks, which effectively improved the speed and accuracy of osteosarcoma MRI images.

From the above research work, it can be seen that image segmentation methods have become increasingly important for disease diagnosis and prognosis. However, as shown in Table 1, existing studies still face many problems in the detection of osteosarcoma MRI images. In particular, it is still difficult to reasonably preserve edge features when segmenting osteosarcoma images. Since images are sensitive to noise, it is necessary to reduce MRI image noise to improve segmentation accuracy. To compensate for segmentation inaccuracy, we present a segmentation method based on edge enhancement from osteosarcoma MRI (OSTransnet). The method uses strategies such as dataset optimization, model segmentation, edge enhancement, and mixed loss functions to improve the accuracy of osteosarcoma segmentation.

## 3. System Model Design

**Figure 1:**
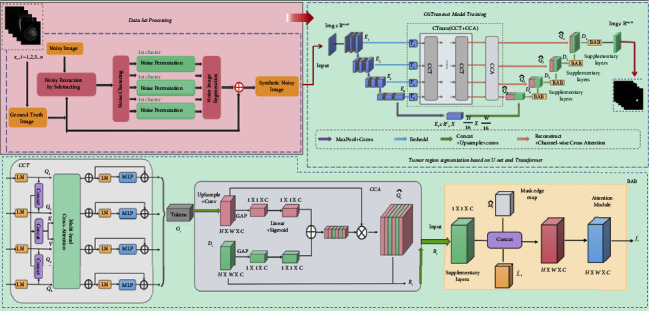
Overall architecture diagram.

The diagnosis and treatment of osteosarcoma present many difficulties in most underdeveloped countries due to financial and technical constraints [58]. Osteosarcoma MRI scans is complex and data-intensive. Manual screening and diagnostic tests, which cost a lot of medical resources and are difficult for clinicians, are extremely difficult to execute [59, 60]. Image processing technology is gradually becoming more frequently employed in disease diagnosis, treatment, and prognosis to aid clinicians in clinical diagnosis and increase disease diagnosis efficiency [61]. In addition, due to the complexity of osteosarcoma MIR images and the diversity of tumors, existing detection methods do not achieve ideal segmentation results [62]. This study offers a segmentation approach (OSTransnet) for osteosarcoma MRI images with edge enhancement features based on Transformer and U-Net, which is primarily intended to assist clinicians in more precisely and rapidly diagnosing osteosarcoma lesions areas by recognizing osteosarcoma MRI pictures. It has been experimentally demonstrated that OSTransnet outperforms the current famous network architecture in segmentation accuracy for the segmentation of osteosarcoma.  Figure 1 depicts the overall layout of this publication.

We construct an edge-enhanced osteosarcoma MRI image segmentation method (OSTransnet), which is mainly divided into two parts: dataset optimization processing and MRI image segmentation model based on U-Net and Transformer with edge-enhanced features. In Section 3.1, we introduced a new data alignment. It is better for the subsequent segmentation and diagnosis of the osteosarcoma lesion region. By taking the optimized image data in 3.1 and feeding it into the segmentation network in 3.2, we can locate the location and extent of the tumor and provide aid to the doctor's decision-making for diagnosis and prediction of the disease.

### 3.1. Dataset Optimization

One of the most important problems in AI-assisted diagnosis systems is the lack of labeled pictures for diagnosing osteosarcoma, despite a large amount of data in MRI images. Deep learning-based models are prone to overfitting if there are insufficient training samples. Data enhancement is an effective way to avoid the overfitting problem. At the same time, osteosarcoma images have the characteristic of being susceptible to noise. It is not feasible to directly discard labeled images that contain noise, and they can also contribute to the model. We introduce a new data alignment method that utilizes the natural noise in authentic noisy images to solve this problem. More training examples with actual content and noise are generated by altering the spatial distribution of natural noise.

**Figure 2:**
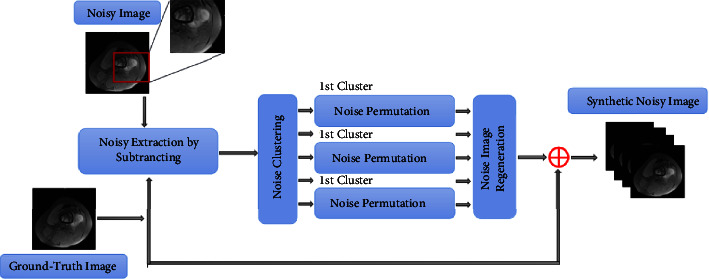
Dataset optimization process.

The  first step is to create noisy picture data by subtracting the validly labeled photos from the corresponding noisy images, as shown in Figure 2. When working with noisy data, the noise clustering technique divides it into groups based on ground-truth intensity values. The places of these noises are then swapped using a random permutation inside each cluster. The displaced image is combined with the accompanying valid, ground-truth labeled image to form a new synthetic noisy MRI image. This is done to limit the impact of noise on segmentation model accuracy while expanding the breadth of data.

In this section, we preprocessed osteosarcoma MRI images. The processed images can not only reduce the waste of ineffective model training but also improve the segmentation performance. Furthermore, these images can be used as a reference for doctors' clinical diagnoses, which can also improve detection accuracy and diagnosis speed. In the next section, we describe the MRI image segmentation process in detail.

### 3.2. Osteosarcoma Image Segmentation

**Figure 3:**
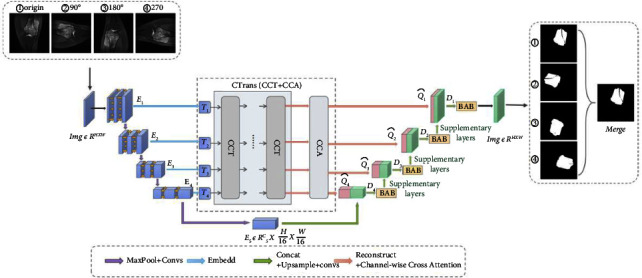
Segmentation model diagram.

The osteosarcoma segmentation model consists of four main parts: U-Net without skip connection mechanism, channeled Transformer module (CTrans), edge enhancement module (BAB), and combined loss function. The general design is shown in Figure 3.

#### 3.2.1. U-Net without Skip Connection Mechanism

U-Net [30] is the most commonly used model for image segmentation in the medical field due to its lightweight properties. Its performance in medical picture segmentation as a traditional encoder-decoder network structure has been outstanding. As a result, the U-Net model is used to segment MRI images in the case of osteosarcoma. The systolic path and the extended path are the two sections that make up the U-Net in general. The systolic path is on the left and functions mostly as an encoder for low-level and high-level characteristics. It is made of two 3 × 3 unfilled convolutional repetitions and follows the conventional construction of a convolutional network. Following that, a 2 × 2 maximum pooling operation and a rectified linear unit (ReLU) are coupled. After each convolution, there is a two-step downsampling process. During each layer's downsampling, the number of feature channels is multiplied by two. The extended path, on the right, is mostly employed as a decoder, combining semantic characteristics to produce the final result. Upsampling the feature map and conducting a 2 × 2 upconvolution are included in each stage of its journey. It halves the number of features to match the relevant feature maps in the associated shrinkage path. Once the features are linked, the osteosarcoma MRI feature map is subjected to a 3 × 3 convolution. Each convolutional output of the feature map must go through ReLU once more.

The feature connection in the original U-net uses a skip connection mechanism. The features in the encoder and decoder stages are incompatible, leading to a semantic gap, which has a certain impact on the segmentation model. To segment osteosarcoma MRI images more accurately, we introduced channel-based transformers (CTrans) instead of U-Net's skip connection. It takes advantage of the transformers and U-Net for cross-fusion of multiscale channel information to achieve effective connection with decoder feature disambiguation. The multiscale exploration of sufficient information of global context bridges the semantic gap and solves the problem of semantic hierarchy inconsistency. Better segmentation results are obtained in this way.

#### 3.2.2. Channeled Transformer Module (CTrans)

**Figure 4:**
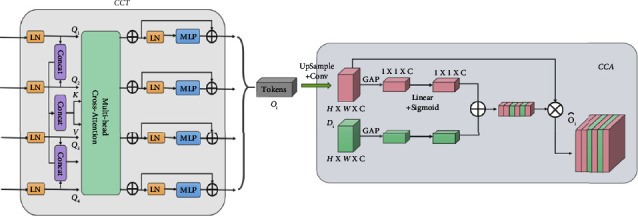
Channeled transformer module (CTrans).

To eliminate semantic delay and integrate encoder features to improve the segmentation effect of osteosarcoma MRI images, a channel conversion module is constructed in this paper, as shown in Figure 4. This is mainly to achieve channel-dependent transformation between the U-Net encoder and decoder. This module consists of two parts: the Channel-wise Cross Fusion Transformer (CCT) and the Channel-wise Cross-Attention (CCA). CCT realizes multilevel coding fusion and CCA is used for decoding fusion. Among them, the extended CCT fusion replaces U-Net with a channel transformer (CTrans).

*(1) CCT: Channel Cross-Merging Transformer for Transforming Encoding Functions*. We present a new channel-based cross-fusion transformer (CCT) that uses long-dependent modeling in the Transformer to fuse multiscale encoder characteristics in osteosarcoma MRI images during segmentation to better fuse multiscale features. The CCT module consists of three parts: multiscale feature embedding, multihead channel cross-attention, and multilayer perceptron. They are described in detail below.

*Multi-scale feature embedding.* We tokenize the osteosarcoma features and restructure them into flattened 2D patch sequences. So that the patch can be mapped to the same region of the encoder at four scales, we set the patch size to *P*, *P*/2, *P*/4, *P*/8, respectively, and use the four skip-connected layer outputs of the multiscale feature embedding *E*_*i*_ ∈ *R*^*HW*/*i*^2^×*C*_*i*_^. We preserve the original channel sizes during this process. The four layers *T*_*i*_(*i*=1,2,3,4), *T*_*i*_ ∈ *R*^*HW*/*i*^2^×*C*_*i*_^ as key values are then connected.

(1) $$\begin{array}{c} T_{\Sigma }=Concat\left(T_{1},T_{2},T_{3},T_{4}\right)\text{.} \end{array}$$

*Multichannel cross-notice module.* This is passed to the multihead channel cross-attention module, which uses multiscale features to refine features at each U-Net encoder level. Then, there is a multilayer perceptron (MLP) with a residual structure that encodes channels and dependencies.

**Figure 5:**
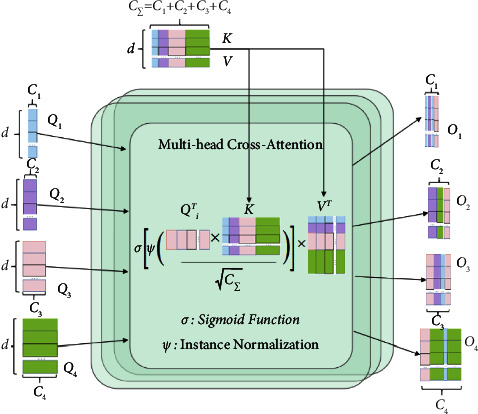
Multihead channel cross-notice module.

The proposed CCT module has five inputs, as shown in Figure 5, with four tokens *T*_*i*_ serving as queries and a connected token *T*_Σ_ serving as keys and values:

(2) $$\begin{array}{c} Q_{i}=T_{i}W_{Q_{i}},K=T_{\Sigma }W_{K},V=T_{\Sigma }W_{V}\text{,} \end{array}$$

where *W*_*Q*_*i*__ ∈ *R*^*C*_*i*_×*d*^, *W*_*K*_ ∈ *R*^*C*_Σ_×*d*^, *W*_*V*_ ∈ *R*^*C*_Σ_×*d*^ is the weight of the different inputs, *d* is the length of the sequence, *Q*_*i*_ ∈ *R*^*C*_*i*_×*d*^, *K* ∈ *R*^*C*_Σ_×*d*^, *V* ∈ *R*^*C*_Σ_×*d*^, the values of the acquaintance matrix *M*_*i*_ and *V* are weighted. and *C*_*i*_(*i*=1,2,3,4) is the size of the channel that skips the connection layer.

(3) $$\begin{array}{c} C_{1}=64,{\;C}_{2}=128,C_{3}=256,C_{4}=512\text{.} \end{array}$$

The cross-attention (CA) mechanism is as follows:

(4) $$\begin{array}{c} {CA}_{i}=M_{i}V^{T}=\sigma \left[\Psi \left(\frac{Q_{i}^{T}K}{\sqrt{C_{\Sigma }}}\right)\right]V^{T}=\sigma \left[\Psi \left(\frac{W_{Q_{i}}^{T}T_{i}^{T}T_{\Sigma }W_{K}}{\sqrt{C_{\Sigma }}}\right)\right]W_{V}^{T}T_{\Sigma }^{T}\text{,} \end{array}$$

where *ψ*(·) and *σ*(·) denote the random normalization and softmax functions, respectively.

We operate attention along the channel axis instead of the patch axis, which is quite different from the original self-attention mechanism. By normalizing the similarity matrix for each instance on the similarity maps, we can smooth down the gradient by using instance normalization. The output after multihead cross-attention in an *N*-head attention condition is computed as follows:

(5) $$\begin{array}{c} {MCA}_{i}=\left({CA}_{i}^{1}+{CA}_{i}^{2}+\cdots +{CA}_{i}^{N}\right)N\text{.} \end{array}$$

In this formula, *N* is the total number of heads.

After that, we use MLP and residual operator to get the following output:

(6) $$\begin{array}{c} O_{i}={MCA}_{i}+MLP\left(Q_{i}+{MCA}_{i}\right)\text{.} \end{array}$$

For simplicity, we omit layer normalization (LN) from the equation. We repeat the operation of formula (6) *L* times to finally form an L-layer transformer. where *N* and *L* are both set to 4. This is mainly because with 4 layers and 4 heads, the model can achieve state-of-the-art segmentation performance on the dataset after experimental validation with 2, 4, 8, and 12 layers based on CCT.

*(2) CCA: Cross-Channel Focus for Feature Synthesis in Decoders*. The channel-based cross-notification module filters and disambiguates the decoder features by channel and information that guide the interrogator features. Its main purpose is to fuse features that are semantically inconsistent between the channel interrogator and the U-Net decoder.

We use the level *i* transformer output *O*_*i*_ ∈ *R*^*C*×*H*×*W*^ and the level *i* decoder feature map *D*_*i*_ ∈ *R*^*C*×*H*×*W*^ as inputs to the global average pooling (GAP) layer, which uses them to incorporate global spatial information and shape attention:

(7) $$\begin{array}{c} M_{i}=L_{1}\cdot \varsigma \left(O_{i}\right)+L_{2}\cdot \varsigma \left(O_{i}\right)\text{,} \end{array}$$

where *ς*(*X*)=1/*H* × *W*∑_*i*=1_^*H*^∑_*j*=1_^*W*^*X*^*k*^(*i*, *j*), *ς*(*X*) ∈ *R*^*C*×1×1^, *L*_1_ ∈ *R*^*C*×*C*^, *L*_2_ ∈ *R*^*C*×*C*^ and being weights of two linear layers and the ReLU operator *δ*(·).

To avoid the effect of dimensionality reduction on channel attention learning, we are constructing channel attention maps with a single linear layer and S-shaped functions, and synthetic vectors are used to recalibrate and excite *O*_*i*_.

With this method, the process of transformer self-control is rethought from the perspective of the channel to close the semantic gap between features through more effective feature fusion and multidimensional channel cross-checking. This enables acquiring more intricate channel dependencies to enhance the functionality of MRI image segmentation models for osteosarcoma.

#### 3.2.3. Edge Enhancement Module (BAB)

**Figure 6:**
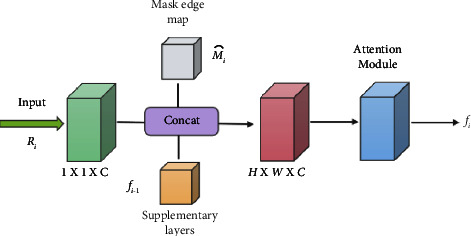
Edge enhancement (BAB) module.

**Figure 7:**
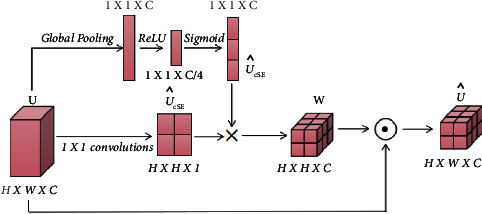
Attention mechanism of BAB module.

In the MRI image segmentation of osteosarcoma, blurred edge segmentation, and partial region missing have been the main problems to be solved, which affect the accuracy of MRI image segmentation to a certain extent. We introduce the edge augmentation block (BAB) to solve this problem, as shown in Figure 6. It focuses more on enhancing the edge information of the lesion region by a mask extraction algorithm and attention mechanism, as shown in Figure 7. Edge enhancement is performed on osteosarcoma MRI images to supplement the missing regions. The BAB module solves the segmentation problem of blurred edges to a certain extent.

The final feature map *D*_1_,*D*_2_,*D*_3_,*D*_4_ of the decoder in the U-Net path is fed to the BAB module as an input layer.

After convolving the input feature map, the mask edge map *M*_*i*_ is obtained by the mask edge extraction algorithm as an important complement to the edge information. The process of the mask edge extraction algorithm can be expressed as follows: traverse each pixel point (*i*, *j*) of the mask, when the traversed pixel value is 0 and the rest of the pixel points in the nine-box grid centered on the pixel point are not all 0, the pixel point is recorded as 0 until all the pixel points of the mask are traversed, and then, the mask edge map *M*_*i*_ is generated.

The feature maps obtained after convolution are connected with the complementary layer feature maps *f*_*i*−1_) obtained from the previous layer after BAB upsampling by channel and input to the attention module to obtain the final prediction.

(8) $$\begin{array}{c} F_{i}=AB\left(d_{3}\left\{c\left[d_{3}\left[c\left(d_{1}\left(R_{i}\right),\hat{M_{i}}\right)\right],f_{i-1}\right]\right\}\right)\text{,} \end{array}$$

where *d*_*s*_(∙) denotes the convolution function, *c*(∙) denotes the join operation, *AB*(∙) denotes the attention module function, and *U* ∈ *R*^*C*×*H*×*W*^ denotes the output.

For the input feature map *U* ∈ *R*^*C*×*H*×*W*^, the feature map *U*_*sCE*_ ∈ *R*^*C*×*H*×*W*^ and vector *U*_*sCE*_ ∈ *R*^1×1×*C*^ are obtained by compressing them on the channel and space, respectively, and the two are multiplied to obtain the weight *W*∈*R*^*C*×*H*×*W*^, which is then multiplied pixel by pixel with the input feature map $\hat{U}$ to obtain the output.

(9) $$\begin{array}{c} \hat{U}=\left(\hat{U_{sCE}}\times \hat{U_{cSE}}\right)⊙U\text{.} \end{array}$$

where × represents direct multiplication after expansion to read and ⊙ represents pixel-by-pixel multiplication.

#### 3.2.4. Combined Loss Functions

Osteosarcoma MRI images often have the problem of class imbalance, which leads to the training being dominated by the class with more pixels. It is challenging to learn the features of the part with fewer pixels, thus, affecting the effectiveness of the network. Therefore, we mostly use the Dice loss function, which measures the overlapping part of the samples, to solve the class imbalance. However, for osteosarcoma, MRI images have the image characteristics of blurred edges, and the Dice loss function cannot focus on the image edge information. So we propose a combined loss function *L*. It combines region-based Dice loss and edge-based Boundary loss, supervised in two different focus dimensions. Dice loss and Boundary loss are defined as follows:

(10) $$\begin{array}{c} L_{Dice}=1-\frac{2\overset{N}{\underset{i=1}{\sum }}\overset{C}{\underset{c=1}{\sum }}g_{i}^{c}S_{i}^{c}}{\overset{N}{\underset{i=1}{\sum }}\overset{C}{\underset{c=1}{\sum }}g_{i}^{c^{2}}+\sum_{i=1}^{N}\overset{C}{\underset{c=1}{\sum }}S_{i}^{c^{2}}}\text{,} \end{array}$$

where *i* denotes each pixel point, *c* denotes the classification, *g*_*i*_^*c*^ denotes whether the classification is correct, and *s*_*i*_^*c*^ denotes the probability of being classified into a certain class.

(11) $$\begin{array}{c} L_{BD}=\int_{\Omega }\phi_{G}\left(\xi \right)S_{\theta }\left(\xi \right)d\xi \text{.} \end{array}$$

If *ξ* ∈ *G*(Goung Truth), then *ϕ*_*G*_(*ξ*)=−*D*_*G*_(*ξ*), and vice versa *ϕ*_*G*_(*ξ*)=*D*_*G*_(*ξ*). Where *ϕ*_*G*_ is the bounded level set representation, *D*_*θ*_(*ξ*) is the distance map of ground truth, and the network's softmax probability output is *S*_*θ*_(*ξ*).

The combined loss function *L* is defined, as shown in (11):

(12) $$\begin{array}{c} L=\alpha L_{Dice}+\beta L_{Dice}\text{,} \end{array}$$

where, parameters *α* and *β* are balance coefficients to balance the effect of area loss and edge loss on the final result.

The loss function *L* combines the region-based Dice loss and the edge-based Boundary loss, allowing the network to focus on both region and edge information. It complements the edge information while ensuring small missing values in the region, thus improving the accuracy of segmentation. As the neural network continues to iterate, the balance coefficients *α* and *β* are updated by self-learning adjustments, prompting the Dice loss to occupy a larger proportion of the first half of the U-Net network. Thus, the U-Net network is relatively more concerned with regional information. Boundary loss pays more attention to edge information, so it occupies a larger proportion of the second half of the edge-attention module. In this paper, a combined loss function is used to play the role of an edge attention module, which realizes attention to regional information without losing edge information. It solves the problems of large missing values and unclear edges in current medical image segmentation.

Not only can our segmentation algorithm accurately segment the tumor region in different slices of osteosarcoma MRI images, but it can also solve the problem of the lesion region's hazy boundary in osteosarcoma MRI pictures. Our model places a greater emphasis on edge information, which is beneficial for precise border segmentation. The final lesion area and segmentation results from the model can help doctors diagnose and treat osteosarcoma. It helps to increase the effectiveness and accuracy of osteosarcoma diagnosis, which lessens the pressure on doctors in many nations to treat osteosarcoma. Additionally, it is crucial for the auxiliary diagnosis, prognosis, and prediction of osteosarcoma disease.

## 4. Experimental Results

### 4.1. Dataset

The Center for Artificial Intelligence Research at a Monash University provided the data for this article [57]. We gathered more than 4,000 MRI osteosarcoma pictures and other index data. To improve the accuracy and robustness of the model segmentation results, we rotated the photos by 90, 180, and 270 degrees before feeding them into the segmentation network. The training set consisted of 80% of the data, whereas the test set consisted of 20% of the data.

### 4.2. Evaluation Metrics

To evaluate the performance of the model, we used the Intersection of Union (IOU), Dice Similarity Coefficient (DSC), Accuracy (ACC), Precision (Pre), Recall (Re), and F1-score (F1) as the measures [63]. These indicators are defined as follows:

(13) $$\begin{array}{c} IOU=\frac{I_{1}\cap I_{2}}{I_{1}\cup I_{2}}\text{,}\\ DSC=\frac{2*\left|I_{1}\cap \left.I_{2}\right|\right.}{\left|I_{1}\right|+\left|I_{2}\right|}\text{,}\\ \begin{array}{c} Acc=\frac{TP+TN}{TP+TN+FP+FN},\\ Pre=\frac{TP}{TP+FP},\\ \begin{array}{c} Re=\frac{TP}{TP+FN},\\ F1=\frac{2\times Pre\times Re}{Pre+Re}, \end{array} \end{array} \end{array}$$

where *I*_1_, *I*_2_ are the predicted and actual tumor areas, respectively. A true positive (*TP*) indicates that the area has been identified as an osteosarcoma area. A true negative (*TN*) indicates that the area is considered normal, although it is also a lesion area. A false positive (*FP*) is normal tissue that has been determined to be tumor-free. A false negative (*FN*) indicates an area predicted to be normal but it is a tumor area [64].

In addition, for comparative experimental analysis, we use the FCN [65], PSPNet [66], MSFCN [48], MSRN [49], U-Net [67], FPN [68], and our proposed OSTransnet algorithms. Below is a quick description of these strategies.

### 4.3. Training Strategy

To improve the robustness of the model and avoid nonsense features, we need to perform data augmentation on the dataset before training. We use natural noise augmentation to increase the dataset by rotating the image.

For the AI model, the rotation of the image is obtained as a new image. To make the mini-row segmentation effect more robust and accurate, we rotated one image by 90, 180, and 270 as data augmentation to finally obtain the segmentation probability as a weighted average of the four image probabilities.

A total of 200 epochs were trained to create a segmentation neural network. In the U-net, a joint training optimization strategy was applied to the convolution and CTrans parameters, and the inferior attention parameters of the two channels were optimized. We first trained the U-net and then the parameters of the OSTransnet using the same data.

### 4.4. Results

**Figure 8:**
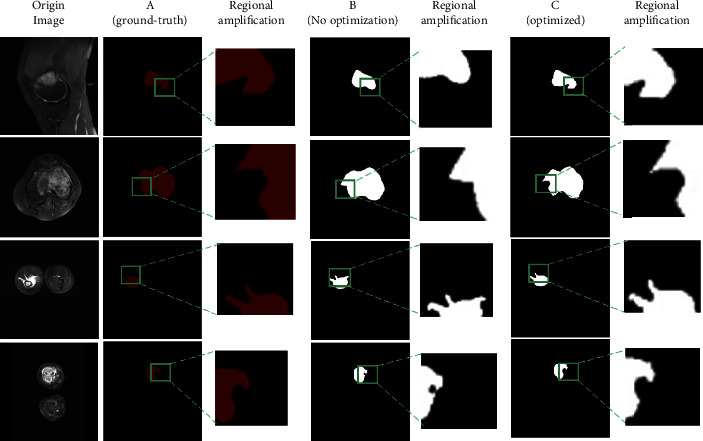
Comparison of the impact of segmentation before and after dataset optimization.

The segmentation effect of the model before and after dataset tuning is shown in Figure 8. Each row has three columns: column A represents the ground truth, column B represents the model's segmentation effect graph without dataset optimization, and column C represents the model's segmentation effect after optimization. In the zoomed-in image of the local area before optimization, as illustrated in column B, partial and erroneous segmentation occurs. After the dataset optimization, the model segmentation results are closer to the real labels, as shown in column C. The completeness and accuracy of the segmentation results can be clearly seen in the enlarged image of the local region. It can be seen that before the dataset is optimized, there is an impact on the segmentation model accuracy due to MRI image noise. After the dataset is optimized, the data augmentation operation using real noise suppresses the influence of noise on the accuracy of the segmentation model to a certain extent and there are significant improvement in segmentation completeness and accuracy. Furthermore, for tumor margins in MRI images, the segmentation effect is significantly improved.

As shown in Table 2, the dataset optimization and edge improvement modules are advantageous in improving the prediction results, demonstrating that optimizing the dataset may considerably improve the OSTransnet border segmentation and improve the results. Preincreased by around 0.5%, F1 increased by roughly 0.3%, IOU increased by roughly 0.7%, and DSC increased by roughly 0.7%. Following segmentation optimization, DSC improved by 1.1%, Pre by 0.2%, Re by 0.5%, F1 by 0.2%, and IOU by 0.8%, respectively.

**Figure 9:**
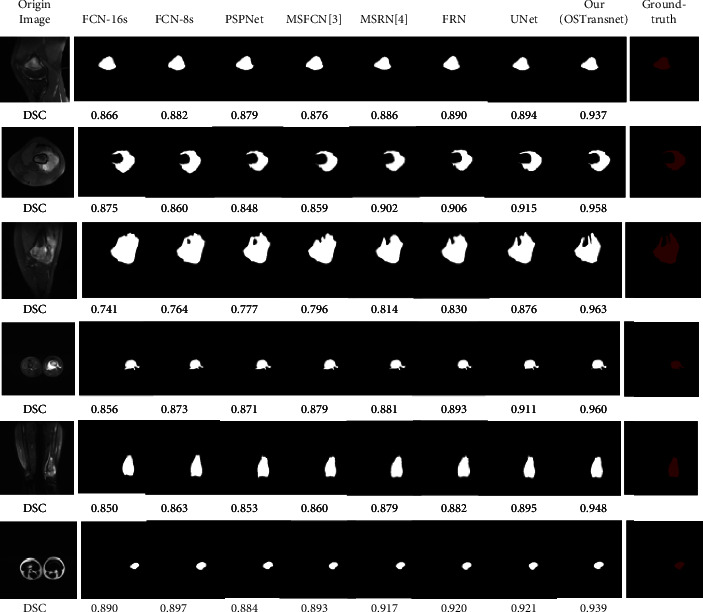
Comparison of the effect of each model on MRI image segmentation of osteosarcoma.

Furthermore, the following Figure 9 shows the effect of each model on the segmentation of osteosarcoma MRI images. We compared the effect plots of FCN-16s, FCN-8s, PSPNet, MSFCN, MSRN, FPN, and U-Net with our OSTransnet segmentation model. Ground-truth segmented images can be used to visually examine the model's segmentation performance. Meanwhile, we chose the DSC metrics. The following 6 osteosarcoma segmentation examples show that OSTransnet can achieve better segmentation results in osteosarcoma MRI image segmentation work. Especially in MRI images with blurred tumor borders, such as the third example with more tumor border segmentation, our method is more accurate and complete in segmentation. For FCN, PSPNet, and MSFCN models, there is an oversegmentation problem.

**Figure 10:**
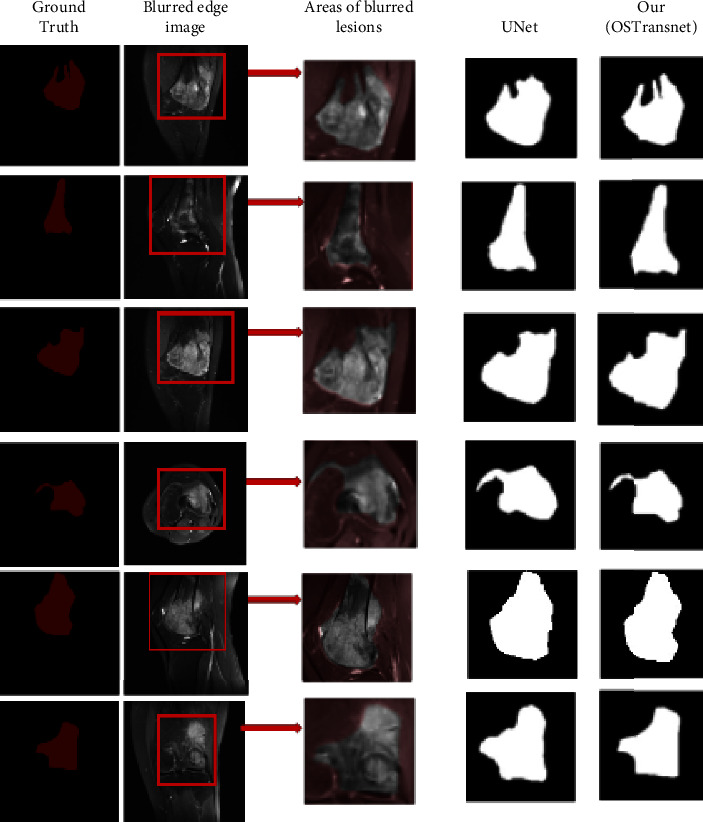
Comparison of edge blur image segmentation effect.

To evaluate the segmentation effect of the model on MRI images with fuzzy edges, we selected six osteosarcoma images with the same fuzzy edge feature as the third example in Figure 9 for detailed comparison. In this paper, we used U-Net, which has the best segmentation effect among many comparison models, and OSTransnet for the comparative analysis of the images. From the detailed comparison in Figure 10, we can intuitively see that our model has a more accurate segmentation effect for the images with the blurred boundaries of the lesion regions. Compared with other contrasting models, our OSTransnet model has greater advantages in boundary blur segmentation due to its unique edge enhancement module and combined loss function. It can be clearly seen that it more effectively and accurately segments the boundary of the lesion area. The OSTransnet model effectively solves the blurred segmentation edge that often occurs in osteosarcoma MRI images.

We quantified the performance of each method in order to further examine the performance of each strategy. Experimental evaluation was performed on the osteosarcoma MRI dataset, and the results are shown in Table 3. The accuracy of the FCN-8s model was the highest, but the performance was poor in several other metrics. In particular, the recall rate was the worst for FCN. The recall rate was only 0.882 for FCN-16s and 0.873 for FCN-8s. The PSPNet model had the lowest IOU at 0.772. The MSFCN and MSRN models showed relatively improved performance. Both models have improved substantially in all metrics, with recall rates reaching 0.9. The U-Net model has the best performance of all the compared methods, with an IOU of 0.867 and a DSC of 0.892. The performance of the OSTransnet model proposed in this paper is the best. It has the highest results in several metrics of DSC, IOU, Recall, and F1. It achieved a DSC value of 0.949, which is about 6.4% better than U-Net. It indicates that the OSTransnet model has better performance in osteosarcoma segmentation.

**Figure 11:**
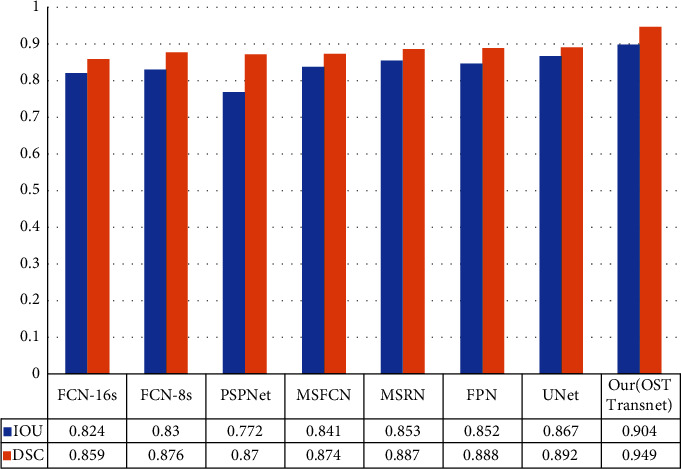
Comparison of DSC and IOU for different methods.

On the osteosarcoma dataset, Figure 11 illustrates the segmentation comparison of different approaches, and we used IOU for numerical comparison with DSC. Our proposed osteosarcoma segmentation model is more accurate, with the DSC metric being 5% higher than the second U-Net and the IOU measure being 4% higher than the second U-Net, according to the data.

**Figure 12:**
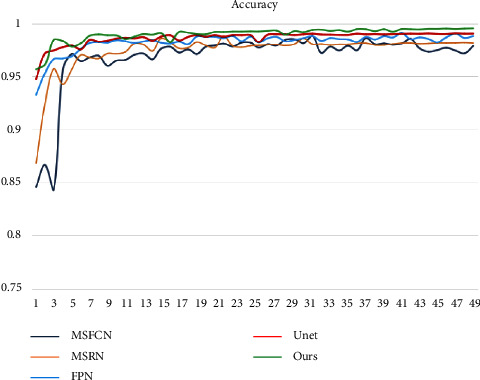
Variation of accuracy for each model.

**Figure 13:**
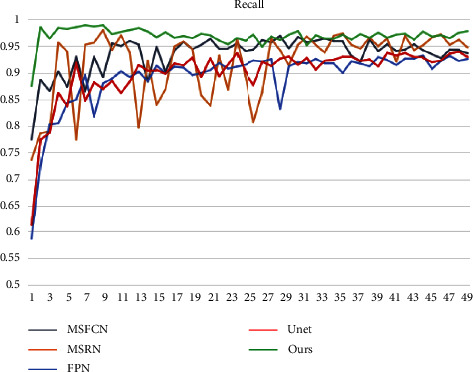
Change in recall of each model.

Figure 12 depicts the accuracy variation of each model. We trained a total of 200 epochs and utilized systematic sampling to select 50 epochs at random (1 epoch randomly selected per 4 epochs) for comparative analysis. You can see that the accuracy of each model begins to stabilize after an average of 50 epochs. Our OSTransnet offers the highest value stability, with 98.7% reliability. The accuracy ranking among the models is OSTransnet > U-Net > FPN > MSRN > MSFCN based on the photos supplied. The recall of MRSN and MSFCN changes substantially throughout the first 120 periods of training, as shown in Figure 13. Except for MSRN, the other models converge to a stable state after that. Overall, the recall rate of our suggested method has been kept as high as possible, ensuring that the risk of missing a diagnosis is minimal.

**Figure 14:**
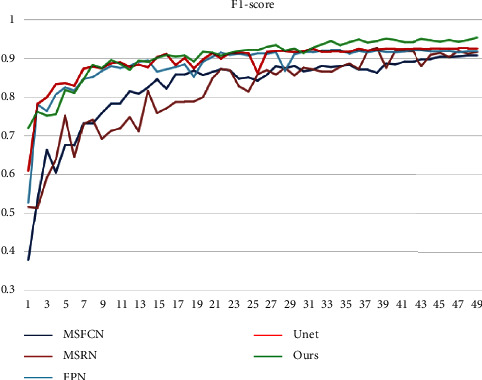
Change of F1-score of each model.

Finally, we used our approach to compare each model's F1-score. The F1 of each model changes, as shown in Figure 14, although our model swings the least in comparison. In addition, when compared to the F1 of other models, our model's F1 is always the greatest. This demonstrates the robustness of our method. We obtained better performance and segmentation results for the osteosarcoma MRI dataset compared to the segmentation results for each of the models in the table. This method can be used to diagnose, treat, and predict osteosarcoma, as well as offer doctors a diagnostic tool for the disease.

### 4.5. Discussion

According to the analysis in Section 4.4, the performance of each model has a large gap in tumor region recognition. On the one hand, the shape and location of osteosarcoma MRI images vary greatly. On the other hand, the osteosarcoma MRI images are limited by the acquisition equipment, resulting in low resolution and high noise. All these have a large impact on the segmentation effect. The use of deeper and more complex networks alone does not improve the segmentation accuracy well. The performance of the FCN model is relatively poor, and it is easy to misclassify normal tissues as tumor regions. Although the performance of the PSPNet model and FPN mode has improved, both have lower recognition accuracy for tumor subtleties and different scales of tumors. Both the MSFCN and MSRN models showed substantial improvements in all metrics, but the performance of these two models still fell short of the ideal due to the heterogeneity of osteosarcoma and the complexity of the MRI image background. The U-net model can better avoid the interference of complex background in MRI images by incorporating contextual information, so it has better segmentation performance and all indexes are better than the other methods in the experiment. However, due to the network architecture, it is not sensitive enough to multiscale tumors and edge details.

Our OSTransnet model has the best segmentation performance. Especially for tumors of different scales and for subtleties between tumors. It achieves better segmentation results for both. This is mainly due to the combination of Transformer and U-Net network models we used. By introducing the Channel Transformer (CTrans) module to replace the jump connection in U-Net. It effectively solves the problem of semantic defects in U-Net, thus completing the identification of tumors at different scales. In addition, we introduce the edge enhancement module (BAB) with a combined loss function. This module can improve the accuracy of tumor boundary segmentation and effectively solve the problem of tumor edge blurring.

However, although this approach abbreviates the semantic and resolution gaps, it still cannot fully capture local information due to the introduction of the channel attention cross-attention model. It still has difficulty completing the identification of tumors at different scales in MRI maps. In addition, the small sample dataset has a large impact on the performance of the model. Overall, the results from Section 4.4 show that our approach has less computational cost and better segmentation performance, achieving a better balance between model effectiveness and efficiency. The superiority of the OSTransnet method can be visualized from Figure 9 and Table 3. Therefore, our method is more suitable for clinical aid in diagnosis and treatment.

## 5. Conclusions

In this study, a U-Net and Transformer-based MRI image segmentation algorithm (OSTransnet) for osteosarcoma with edge correction is proposed. Dataset optimization, model segmentation, edge improvement, and a combined loss function are all part of the strategy. The method outperforms other existing methods and has good segmentation performance, according to the findings of the experiments. In addition, we visualized the segmentation findings for data processing, which can aid clinicians in better identifying the osteosarcoma lesion location and diagnosing osteosarcoma.

With the development of image processing techniques, we will add more information to the method, enabling us to design a multiscale segmentation method. This will help us to better address segmentation errors caused by slight gray-scale differences between tumor tissue and surrounding tissue, as well as improve the accuracy of segmentation.

## Figures and Tables

**Table 1:** Comparison of different auxiliary diagnostic methods for osteosarcoma.

Detection object	Literature	Technology involved	Application advantages	Limitation
CT image	Literature [48]	Image normalization, CNN	Make sure to capture global and local image features	Segmentation performance is limited in the face of small tumor regions
	Literature [49]	Residual network	Realize the extraction of image shape and semantic features	
	Literature [51]	U-Net, channel attention module	Prevents loss of detail caused by multiple encodings and subsampling	

Pathological image	Literature [44]	Regularization model, CNN	Differentiate between live tumors, necrotic tumors, and nontumors	Pathological evaluation of tissue samples is prone to interobserver variability and is highly subjective. Some of the features used as input to automated machine learners depend on the features identified by the pathologist and require higher costs
	Literature [45]	Siamese network, FCN	Solve the problem of small data overfitting	
	Literature [46]	CNN, transfer learning, VGG19	Automatic classification of tissue images to predict patient conditions	
	Literature [52]	CNN	Prognostic factors predicting survival in osteosarcoma, assessing necrosis rates within a variable range	
	Literature [54]	GAN, F–HHO algorithm	Detect and segment the extracted image features	
	Literature [55]	Deep learning	Lays the groundwork for an automated process for obtaining tumor prediction maps from raw images	
	Literature [56]	Binary arithmetic optimization	Three types of surviving tumor, nonsurviving tumor and nontumor are distinguished	High computational cost and slow system speed

F-FDG PET image	Literature [52]	CNN	To compare the use of different methods in predicting the effect of neoadjuvant chemotherapy	The data source is relatively single

Diffusion weighted imaging	Literature [54]	CNN	Precise localization of lesions in patients with osteosarcoma	The sample size is small.

MRI	Literature [53]	Support vector machines	Predicting a patient's chemotherapy response before treatment	Not validated for large scale data
	Literature [47]	K-means, chan–vese segmentation	High precision segmentation	The complexity of the model is high
	Literature [57]	Mean-teacher, SepUNet, CRF	Tumor region segmentation	Segmentation accuracy is limited

**Table 2:** Comparison of OSTransnet performance under different conditions.

Model	IOU	DSC	Pre	Re	F1
Our (OSTransnet) No optimization + BAB	0.889	0.931	0.917	0.974	0.946
Our (OSTransnet) No BAB	0.896	0.938	0.922	0.976	0.949
**Our (OSTransnet)**	**0.904**	**0.949**	**0.924**	**0.981**	**0.951**

**Table 3:** Performance comparison of different methods on the osteosarcoma dataset.

Model	IOU	DSC	Pre	Re	F1
FCN-16s	0.824	0.859	0.922	0.882	0.900
FCN-8s	0.830	0.876	**0.941**	0.873	0.901
PSPNet	0.772	0.870	0.856	0.888	0.872
MSFCN	0.841	0.874	0.881	0.936	0.906
MSRN	0.853	0.887	0.893	0.945	0.918
FPN	0.852	0.888	0.914	0.924	0.919
U-Net	0.867	0.892	0.922	0.924	0.923
Our (OSTransnet)	**0.904**	**0.949**	0.924	**0.981**	**0.951**

## Data Availability

Data used to support the findings of this study are currently under embargo while the research findings are commercialized. Requests for data, 12 months after the publication of this article, will be considered by the corresponding author. All data analyzed during the current study are included in the submission.
